# Ubiquitin signaling in pancreatic ductal adenocarcinoma

**DOI:** 10.3389/fmolb.2023.1304639

**Published:** 2023-12-20

**Authors:** Shengnan Lv, Jian Zhang, Xinyu Peng, Huan Liu, Yan Liu, Feng Wei

**Affiliations:** ^1^ Department of Hepatobiliary and Pancreatic Surgery, General Surgery Center, The First Hospital of Jilin University, Changchun, Jilin, China; ^2^ Key Laboratory of Jilin Province for Zoonosis Prevention and Control, Changchun Veterinary Research Institute, Chinese Academy of Agricultural Sciences, Changchun, China

**Keywords:** pancreatic ductal adenocarcinoma, ubiquitination, ubiquitin–protein ligases, deubiquitinating enzymes, proteolysis-targeting chimeras

## Abstract

Pancreatic ductal adenocarcinoma (PDAC) is a highly lethal malignant tumor of the digestive system, characterized by rapid progression and being prone to metastasis. Few effective treatment options are available for PDAC, and its 5-year survival rate is less than 9%. Many cell biological and signaling events are involved in the development of PDAC, among which protein post-translational modifications (PTMs), such as ubiquitination, play crucial roles. Catalyzed mostly by a three-enzyme cascade, ubiquitination induces changes in protein activity mainly by altering their stability in PDAC. Due to their role in substrate recognition, E3 ubiquitin ligases (E3s) dictate the outcome of the modification. Ubiquitination can be reversed by deubiquitylases (DUBs), which, in return, modified proteins to their native form. Dysregulation of E3s or DUBs that disrupt protein homeostasis is involved in PDAC. Moreover, the ubiquitination system has been exploited to develop therapeutic strategies, such as proteolysis-targeting chimeras (PROTACs). In this review, we summarize recent progress in our understanding of the role of ubiquitination in the development of PDAC and offer perspectives in the design of new therapies against this highly challenging disease.

## 1 Introduction

Ubiquitination is one of the best-studied PTMs among more than 200 PTMs reported and is widely present in various proteins that participate in the life process of eukaryotic cells (Song and Luo, 2019). It is a cascade reaction process that starts with the ubiquitin-activating enzyme E1, which catalyzes ATP-dependent ubiquitin activation and the formation of a thioester bond between the C-terminal end of ubiquitin and cysteine on E1. In turn, ubiquitin is transferred to ubiquitin-conjugating enzyme E2 via a similar thioester linkage. Finally, E3s recruit substrate proteins and then transfer ubiquitin to them. E3s are divided into three main families based on the presence of specific functional domains and the mechanism of catalysis: RING E3s, HECT E3s, and RBR E3s.

**FIGURE 1:**
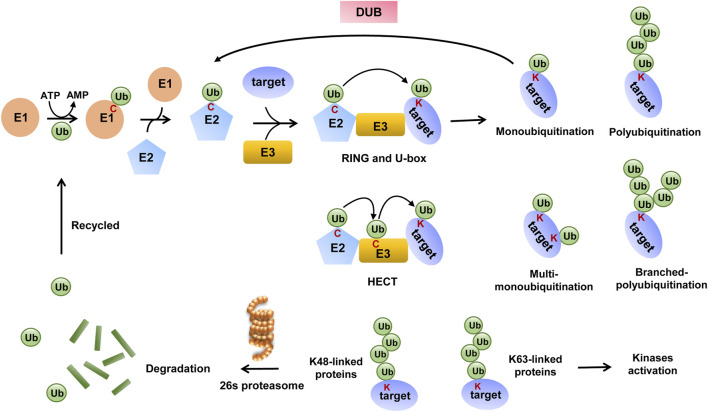
Mechanism of ubiquitination and deubiquitination.

RING E3s are characterized by the presence of a zinc-binding domain or a U-box domain, which orients the ubiquitin-charged E2 to the substrate and directly induces ubiquitin transfer. HECT and RBR E3s both mediate ubiquitin transfer through a two-step reaction, in which ubiquitin is first transferred to a catalytic cysteine on E3 and then transferred from E3 to the substrate (Hershko and Ciechanover, 1998; Berndsen and Wolberger, 2014; Morreale and Walden, 2016; Zheng and Shabek, 2017). The ubiquitination process is shown in Figure 1. Some studies elucidate the existence of E4 enzymes, which act as ubiquitin chain-extending factors and convert monoubiquitination to polyubiquitination (Koegl et al., 1999).

Ubiquitin (Ub) has seven lysine residues (K6, K11, K27, K29, K33, K48, and K63), one methionine (Met) residue at the N-terminal end, and one glycine (Gly) residue at the C-terminal end; therefore, ubiquitination is divided into nine modes accordingly and also named according to the residue of ubiquitin that binds to the substrate lysine residue. Apart from this, when ubiquitin binds to the substrate alone, the ubiquitination type is called monoubiquitination, and if several ubiquitins link with each other and then bind to the substrate, it is called polyubiquitination (Schlesinger and Goldstein, 1975; Kwon and Ciechanover, 2017). Different modes of ubiquitination mediate unique functions, with modification by K48-type poly-Ub chains mainly mediating substrate protein degradation via the 26S proteasome (Finley, 2009), and poly-Ub chains linked by K63 mediating kinase activation or promoting the intracellular transport of modified proteins (Sun et al., 2019).

As a major protease for substrate degradation, a targeting signal must be present for the substrates to be processed by the 26S proteasome, which refers to the polyubiquitin chain *in vivo*. The 26S proteasome has specific ubiquitin receptors, and each receptor is chain-preferred, with the intrinsic receptor preferring K48-linked chains. The ubiquitin receptor leads substrates with K48-linked chains to 26S proteasome, then substrate proteins are degraded, and then dissociated ubiquitins are recycled again for ubiquitination modification systems (Collins and Goldberg, 2017; Bard et al., 2018). Meanwhile, ubiquitination is a dynamic process as some K48-linked polyubiquitinated substrates can escape degradation by the 26S proteasome once ubiquitins are removed from the substrates by several distinct families of DUBs (Leznicki and Kulathu, 2017; Harrigan et al., 2018).

Increasing evidence has identified that E3 and DUBs are able to regulate tumorigenesis and progression by specifically tuning targeting substrates ubiquitination and deubiquitination, thus regulating many vital signaling pathways in different cancer types. However, their roles in PDAC have not been integrated and analyzed yet. Herein, we summarize the major findings on the functions of these enzymes in regulating PDAC progression and draw the network among the current substrates, molecular pathways, and phenotype profiles mediated by E3 and DUBs, and all of them mentioned in this article are listed separately in Table 1 and Table 2.

**TABLE 1:** Summary of E3s reported in PDAC.

Enzyme	Phenotype	Influence on PDAC	Substrate	Influence on substrates	Pathway	Influence on pathways	References
**CBL**	Drug resistance	Suppressor	EGFR	Degradation			Jang et al. (2020); Kadera et al. (2015)
**CFHR**	Proliferation	Suppressor	Plk1	Degradation			Zhu et al. (2021a)
**CUL2**	Proliferation/metastasis	Suppressor	HIF-1α	Degradation			Meng et al. (2022)
**CUL4B**	EMT	Promoter	H2A	Degradation			Leng et al. (2021)
**FATS**	M1 polarization	Suppressor			NF-κB	Inhibition	Zhang et al. (2021c)
**FBXL14**	EMT	Suppressor	Snail	Degradation	Snail	Inhibition	Song et al. (2018)
**FBXO22**	Proliferation	Promoter	LATS	Degradation	LATS	Inhibition	Ma et al. (2023)
**FBXO28**	Proliferation	Promoter	SMARCC2	Degradation			Zhang et al. (2021a); Liu et al. (2023a)
**FBXO45**	Proliferation	Promoter	USP49	Degradation			Zhang et al. (2021a); Wu et al. (2022a)
**FBXW7**	Aerobic glycolysis	Suppressor	MYC	Degradation			Qin et al. (2019)
**FBXW7**	Autophagy/proliferation	Suppressor	SHOC2	Degradation	mTOR	Promotion	Xie et al. (2019)
**FBXW7**	Genomic stability	Suppressor	XRCC4	Degradation			Zhang et al. (2016)
**GID2**	Apoptosis	Promoter					Deng et al. (2023)
**LZTR1**	Proliferation	Suppressor	Ras	Degradation			Palanivel et al. (2022)
**MG53**	Proliferation	Suppressor	cyclin D1	Degradation	Cyclin D1	Inhibition	Ren et al. (2022)
**MIB1**	Proliferation	Promoter	ST7	Degradation			Zhang et al. (2021b)
**MKRN1**	Proliferation	Promoter	P53	Degradation			Liu et al. (2019)
**MURF1**	Muscle contraction	Suppressor					Neyroud et al. (2023)
**NEDD4**	Proliferation	Promoter	PTEN	Degradation	PTEN	Promotion	Su et al. (2017)
**NEDD4L**	Autophagy/proliferation	Promoter	ULK1	Degradation			Lee et al. (2020b)
**NEDD4L**	Ferroptosis	Promoter	LTF	Degradation			Wang et al. (2020)
**PRPF19**	Proliferation	Suppressor	PTPN11	Degradation			Zheng et al. (2016)
**RNF2**	Apoptosis	Suppressor	H2A	Degradation			He et al. (2019)
**RNF43**	Proliferation	Suppressor					Hosein et al. (2022)
**SAG**	Proliferation	Promoter			mTOR	Inhibition	Zhang et al. (2020)
**SCF**	EMT	Suppressor	SMAD4	Degradation	TGF-β	Inhibition	Wan et al. (2005)
**SIAH**	Proliferation	Suppressor					Schmidt et al. (2007)
**Skp2**	EMT	Promoter			TGF-β	Promotion	Wang et al. (2022)
**Skp2**	Proliferation	Promoter	CDKN1B	Degradation			Singh et al. (2018)
**Smurf1**	EMT	Promoter	RhoA	Degradation	TGF-β	Promotion	Zheng et al. (2022)
**Smurf2**	EMT	Promoter			TGF-β	Inhibition	Zhang et al. (2015)
**SPOP**	Stemness	Suppressor	NANOG	Degradation			Tan et al. (2019)
**STIP1**	Metastasis	Suppressor	MZF1	Degradation			Hu et al. (2020)
**TRAF5**	Stemness	Suppressor	NF-κB p65	Degradation	NF-κB	Inhibition	Li et al. (2021a)
**TRIM15**	Lipid metabolism/metastasis	Promoter	APOA1	Degradation			Sun et al. (2021b)
**TRIM21**	Amino acid metabolism	Suppressor	BCAT2	Degradation			Li et al. (2020d)
**TRIM21**	Apoptosis	Promoter	DUSP2	Degradation	AKT	Promotion	Zhang et al. (2023a)
**TRIM21**	EMT	Suppressor	Snail	Degradation	Snail	Inhibition	Liu et al. (2023b)
**TRIM29**	Proliferation	Promoter			YAP1	Promotion	Deng et al. (2021)
**TRIM29**	Stemness	Promoter	ISG15	Degradation			Sun et al. (2020)
**TRIM47**	Aerobic glycolysis	Promoter	FBP1	Degradation			Li et al. (2021c)
**UBR2**	Muscle atrophy	Promoter					Wu et al. (2022b)
**UBR4**	Drug resistance	Suppressor	EZH2	Degradation			Sun et al. (2021a)
**UBR5**	Aerobic glycolysis	Promoter	C/EBPα	Degradation			Chen et al. (2021b)
**UBR5**	Metastasis	Promoter	CAPZA1	Degradation			Li et al. (2021b)
**YOD1**	Metastasis	Promoter					Zhang et al. (2022)
**ZFP91**	Drug resistance	Promoter			β-Catenin	Promotion	Tang et al. (2021)
**ZFP91**	Proliferation	Promoter					Hanafi et al. (2021)
**ZNRF1**	Drug resistance	Suppressor	CAV1	Degradation			Hu et al. (2022)
**β-TRCP1**	Stemness/drug resistance	Suppressor	NRF2	Degradation			Yang et al. (2020b)

**TABLE 2:** Summary of DUBs reported in PDAC.

Enzyme	Phenotype	Influence on PDAC	Substrate	Influence on substrates	Pathway	Influence on pathways	References
**USP10**	Aerobic glycolysis	Promoter	PGK1	Stabilization			Quan et al. (2023)
**USP25**	Aerobic glycolysis	Promoter	HIF-1α	Stabilization			Nelson et al. (2022)
**USP21**	Amino acid metabolism	Promoter					Hou et al. (2021)
**BAP1**	Apoptosis	Promoter	H2A	Stabilization			He et al. (2019)
**USP8**	Cytotoxic T-cell activation	Promoter	PD-L1	Stabilization			Yang et al. (2023)
**USP7**	DNA methylation	Promoter	FBP1	Stabilization			Cheng et al. (2022)
**USP7**	DNA methylation	Promoter	DNMT1	Stabilization			Cheng et al. (2015)
**USP49**	Drug resistance	Suppressor	FKBP51	Stabilization	AKT	Promotion	Luo et al. (2017)
**USP9X**	Drug resistance	Promoter					Ma et al. (2018)
**USP17L2**	Drug resistance	Promoter	Nrf2/YAP	Stabilization	YAP	Promotion	Grattarola et al. (2021)
**OTUD1**	Drug resistance	Promoter	Nrf2/YAP	Stabilization	YAP	Promotion	Grattarola et al. (2021)
**USP18**	EMT	Promoter	Notch1	Stabilization			Feng et al. (2020)
**USP27X**	EMT	Promoter	Snail	Stabilization			Lambies et al. (2019)
**BAP1**	Genomic stability	Suppressor					Perkail et al. (2020)
**USP10**	M2 polarization	Promoter	YAP1	Stabilization	YAP	Promotion	Liu et al. (2022)
**USP14**	Metastasis	Promoter	TAZ	Stabilization	TAZ	Promotion	Zhao et al. (2023)
**USP33**	Metastasis	Promoter	TGFBP2	Stabilization	TGF-β	Promotion	Liu et al. (2023c)
**USP5**	Metastasis	Promoter	WT1	Stabilization			Li et al. (2020c)
**USP9X**	Metastasis	Suppressor					Perez-Mancera et al. (2012)
**OTUB1**	Metastasis	Promoter	FOXM1	Stabilization			Zhang et al. (2023b)
**USP22**	Proliferation	Suppressor	PTEN	Stabilization	PTEN	Promotion	Su et al. (2017)
**USP22**	Proliferation	Promoter			Akt/mTOR	Promotion	Ning et al. (2014)
**USP28**	Proliferation	Promoter	FOXM1	Stabilization			Chen et al. (2021a)
**USP39**	Proliferation	Promoter	NAT10	Stabilization			Feng et al. (2022)
**USP7**	Proliferation	Promoter	β-Catenin	Stabilization	β-Catenin	Promotion	Li et al. (2020a)
**USP9X**	Proliferation	Suppressor	LATS	Stabilization	LATS	Promotion	Toloczko et al. (2017)
**USP9X**	Proliferation	Suppressor					Pal et al. (2018)
**CSN5**	Proliferation	Promoter	β-Catenin	Stabilization	β-Catenin	Promotion	Ma et al. (2021)
**BAP1**	Proliferation	Suppressor	LATS	Stabilization	LATS	Promotion	Lee et al. (2020a)
**OTUD7B**	Proliferation/metastasis	Promoter	EGFR	Stabilization			Lei et al. (2019)
**USP21**	Stemness	Promoter	TCF7	Stabilization	β-Catenin	Promotion	Hou et al. (2019)
**USP10**	Stemness/EMT	Promoter	SOX21	Stabilization			Yu et al. (2022)
**USP44**	Treg differentiation	Promoter	FOXP3	Stabilization			Yang et al. (2020a)

## 2 How E3s and DUBs influence various phenotypes of PDAC

### 2.1 E3s and DUBs regulate cell proliferation in PDAC

Cell proliferation is one of the most important and widely studied phenotypes in PDAC mediated by multiple mechanisms. Among these mechanisms, *KRas* gene mutations account for approximately 90% of PDAC, and mutant KRas proteins maintain an activated state and interact with guanosine triphosphate (GTP), thereby leading to the consistent activation of downstream pathways involved in cell growth and proliferation, such as MAPK and PI3K (Ryan and Corcoran, 2018). Importantly, several E3 ligases are found to be crucial regulators of these pathways. Ring finger protein 43 (RNF43), as a crucial tumor suppressor, inhibits *Kras* ^*G12D*^-dependent tumorigenesis in PDAC cells through single-cell sequencing analysis of KPC (LSL-*Kras* ^*(+/G12D)*^; LSL-*Trp53* ^*(+/R172H)*^; *Pdx1-Cre*) mice (Mishra et al., 2020; Hosein et al., 2022). Leucine-zipper-like transcription regulator 1 (LZTR1) is able to inhibit tumor cell proliferation by degrading Ras proteins (Palanivel et al., 2022). Seven in absentia (SINA) is an important anti-Ras target in *Drosophila*, and silencing the human seven in absentia homolog (SIAH) inhibits Ras-mediated tumorigenesis (Schmidt et al., 2007).

The Hippo pathway plays a negative role in tumorigenesis and cell proliferation, specifically through a series of phosphorylation events of mammalian ste20-like kinases (MST) and large tumor suppressor (LATS), ultimately leading to the phosphorylation of Yes1-associated protein (YAP) and tafazzin (TAZ) (Ma et al., 2019), which finally stay within the cytoplasm and lose their growth promotion effects. Several molecules in this axis can be regulated by E3s and DUBs. BAP1 is identified to inhibit tumor proliferation by stabilizing LATS (Lee et al., 2020a), while tripartite motif-containing 29 (TRIM29) directly binds to YAP1 and avoids YAP1 from ubiquitinated degradation (Deng et al., 2021). F-box protein 22 (FBXO22) was found to be significantly upregulated in tumor tissues of 106 PDAC patients with poor prognosis by causing ubiquitin-dependent degradation of LATS, thus inhibiting the Hippo pathway (Ma et al., 2023).

E3 and DUBs also regulate the Wnt/β-catenin pathway that accelerates cell proliferation and epithelial–mesenchymal transition (EMT) (Ram Makena et al., 2019; Park et al., 2020). Both ubiquitin-specific peptidase 7 (USP7) and COP9 signalosome subunit 5 (CSN5) mediate the deubiquitination and stabilization of β-catenin, thus promoting PDAC proliferation (Li et al., 2020a; Ma et al., 2021). USP22 facilitates the G1/S-phase transition by promoting β-catenin nuclear localization and further enhancing the transcription of forkhead box M1 (FOXM1) (Ning et al., 2014), which, in turn, promotes nucleus β-catenin trans-activation and activates the Wnt/β-catenin pathway. Interestingly, another study found that USP28 can deubiquitinate and stabilize FOXM1, thus promoting cell proliferation through the Wnt/β-catenin pathway (Chen et al., 2021a). These data collectively indicate a positive feedback loop between FOXM1 and the Wnt/β-catenin pathway. USP22 also inhibits tumor proliferation via contributing to the deubiquitination of PTEN (Ren et al., 2022). Therefore, there is discrepancy in the function of USP22, as compared with that mentioned previously, for which our explanation is that different cell lines, namely, SW1990 or PANC1, were used in these two studies, with SW1990 derived from splenic metastasis and PANC1 from the head of the pancreas. The contradictory results indicate different pathways play the dominant role in different stages of PDAC. In addition, mitsugumin 53 (MG53) decelerates the G1/S transition by promoting K48-linked ubiquitination of cyclin D1 and proteasomal degradation (Fang et al., 2023).

Moreover, several important hubs of proliferation-associated pathways are also regulated by E3s and DUBs. S-antigen visual arrestin (SAG) (Zhang et al., 2020) promotes mTORC1 inactivation (Tan et al., 2022) and nuclear factor erythroid 2-related factor 2 (Nrf2) activation. Zinc finger protein 91 (ZFP91) promotes various oncogenic pathways, such as NF-κB and HIF-1α (Hanafi et al., 2021), and neural precursor cell-expressed developmentally downregulated 4-like protein (NEDD4) (Su et al., 2017) promotes PTEN degradation; all of these molecules promote the proliferation of PDAC cells.

In addition to these classical tumor regulatory pathways, E3s and DUBs also influence other promoter or suppressor proteins. Based on the public databases, FBXO22, FBXO28, and FBXO45 are highly expressed in PDAC and correlated with poor prognosis in patients (Zhang et al., 2021a). FBXO28 contributes to the proliferation of PDAC by promoting ubiquitin-mediated degradation of SMARCC2 (Liu et al., 2023a), and FBXO45 facilitates cell proliferation by ubiquitinating USP49 through binding to its SPRY domain (Wu et al., 2022a). In addition, S-phase kinase-associated protein 2 (Skp2) specifically degrades cyclin-dependent kinase inhibitor 1B (CDKN1B) (Singh et al., 2018), and mindbomb homolog 1 (MIB1) enhances the ubiquitin-mediated degradation of suppression of tumorigenicity 7 (ST7) (Zhang et al., 2021b); both Skp2 and MIB1 promote tumor proliferation. On the contrary, some enzymes attenuate tumor proliferation. The complement factor H-related (CFHR) protein family induces the ubiquitination of polo-like kinase 1 (PLK1) at lysine 209 (Zhu et al., 2021a), BRCA1-associated protein 1 (BAP1) enhances genomic stability (Perkail et al., 2020), and USP9X is found to restrain tumor growth in the patient-derived tumor xenograft (PDX) model (Pal et al., 2018). Collectively, these E3s and DUBs either promote or suppress cell proliferation in PDAC.

### 2.2 E3s and DUBs regulate cell death in PDAC

Cell death includes apoptotic and non-apoptotic pathways, and the latter includes autophagy, necrosis, ferroptosis, and pyroptosis, according to different intracellular and extracellular environments. Among these, the most studied E3- and DUB-associated cell death in PDAC is apoptosis.

TRIM21 inhibits tumor cell apoptosis by promoting ubiquitin-dependent degradation of dual-specificity phosphatase 2 (DUSP2) (Zhang et al., 2023a). High expression of glucose-induced degradation-deficient subunit 2 (GID2) is associated with a higher stage and poorer prognosis of PDAC since GID2 inhibits apoptosis by suppressing the ubiquitination of CDKN3 and enhancing its stability (Deng et al., 2023). Conversely, ring finger protein 2 (RNF2) contributes to apoptosis by promoting the monoubiquitination of histone H2A at lysine 119, whereas BAP1 promotes the deubiquitination of H2A. However, BAP1 deficiency does not influence RNF2-dependent apoptosis (He et al., 2019). It is reported that F-box and WD repeat domain-containing 7 (FBXW7) promotes the degradation of SHOC2 that could selectively bind to Raptor and competitively impede the Raptor–mTOR combination. Thus, SHOC2 prevents the inactivation of mTORC1 and inhibits autophagy. Therefore, FBXW7 plays a critical role as an autophagy promoter, thus suppressing cell proliferation and survival in PDAC (Xie et al., 2019).

Intriguingly, NEDD4L downregulates autophagy through inducing ubiquitinated degradation of ULK1, while, differing from FBXW7-induced autophagy, NEDD4L-mediated autophagy promotes tumor proliferation (Lee et al., 2020b), which reflects the “contradictory effect” of autophagy on cell death or growth distinguished from contexts and stages of cancer (Li et al., 2020b). There remains a great challenge to clarify the inherent regulating mechanism between autophagy and tumor cell survival. In addition, NEDD4L is also found to protect tumor cells from undergoing ferroptosis by promoting the ubiquitin-mediated degradation of lactotransferrin (LTF) (Wang et al., 2020). Until now, there is no more study on identifying the role of E3 and DUBs in pyroptosis and necrosis of PDAC.

### 2.3 E3s and DUBs in PDAC cell stemness

Stemness is one of the most malignant hallmarks of tumor cells. Its characteristics are considered to be closely correlated with metastasis and chemoresistance in PDAC and could also be regulated by E3 and DUBs. TNF receptor-associated factor 5 (TRAF5) inhibits cell stemness by promoting ubiquitination degradation of NF-κB p65 and further impeding activin A secretion (Li et al., 2021a). Speckle-type BTB/POZ protein (SPOP) also inhibits cell stemness characteristics by enhancing ubiquitin-mediated degradation of the stem cell marker NANOG (Tan et al., 2019). Conversely, USP21 facilitates cell stemness of PDAC by leading to the deubiquitination of transcription factor 7 (TCF7) (Hou et al., 2019), while knockdown of TRIM29 suppresses cell stemness by promoting the degradation of interferon-stimulated gene 15 (ISG15) (Sun et al., 2020). Cullin 4B (CUL4B) enhances cell stemness by forming a complex with sirtuin 1 (SIRT1), induces the monoubiquitination of H2A, and epigenetically represses the transcription of some tumor suppressors, such as *FOXO3* and *GRHL3* (Leng et al., 2021).

### 2.4 E3s and DUBs mediate drug resistance in PDAC

Chemotherapy resistance is a great challenge in clinical PDAC treatment. Gemcitabine (GEM), as the most popular chemotherapeutic drug, inevitably faces a wide range of resistance. Recombinant ubiquitin–protein ligase E3 component n-recognin 4 (UBR4) inhibits GEM resistance in PDAC by binding to the enhancer of zeste homolog 2 (EZH2) through its N-terminal domain and leading to the ubiquitination degradation of EZH2, whereas histone acetyltransferase 1 (HAT1) can competitively bind to UBR4 and thus stabilize EZH2 (Sun et al., 2021a). USP49 enhances GEM sensitization by promoting the deubiquitination of FK506-binding protein 51 (FKBP51) and further dephosphorylates AKT (Luo et al., 2017). USP17L2 and OTU domain-containing protein 1 (OTUD1) accelerate GEM resistance by upregulating the levels of Nrf2 and YAP (Grattarola et al., 2021). Meanwhile, PDAC patients with high-level ZFP91 typically have stronger drug resistance and shorter patients’ overall survival (OS), while knockdown of ZFP91 enhances the sensitivity of PDAC to GEM (Tang et al., 2021). USP9X is significantly elevated in GEM-resistant cells, while inhibiting USP9X with WP1130 would improve the sensitivity of PDAC cells to GEM (Ma et al., 2018).

Additionally, GEM is reported to suppress epidermal growth factor receptor (EGFR) by stimulating the expression of casitas B-lineage lymphoma (CBL) that promotes ubiquitination degradation of EGFR and inhibits tumor progression (Jang et al., 2020). Meanwhile, another study further elucidated that ablation of CBL prevents the degradation of EGFR, which leads to GEM resistance of PDAC, and the application of erlotinib (an EGFR-targeting antitumor agent) counteracts this consequence (Kadera et al., 2015). This emphasizes the importance of CBL in GEM-induced EGFR-dependent resistance.

### 2.5 E3s and DUBs regulate EMT in PDAC

EMT means the transformation from epithelial cells to mesenchymal cells, a process that gifts tumor cells the ability to invade and migrate. The main characteristics of EMT involve the downregulation of epithelial cell markers, upregulation of mesenchymal cell markers, loss of intercellular adhesion junctions and tight junctions, and alteration of cell morphology and cytoskeletal remodeling.

Snail is a critical EMT-driving transcription factor and confers tumor metastatic properties by directly inhibiting the expression of E-cadherin (Manfioletti and Fedele, 2022). Several E3s and DUBs broadly participate in the Snail-mediated EMT process. For instance, TRIM21 binds to Snail and induces its ubiquitin-dependent degradation, whereas CD73 competitively targets TRIM21 to prevent Snail from degradation (Liu et al., 2023b). F-box and leucine-rich repeat protein 14 (FBXL14) promote the ubiquitin-mediated degradation of Snail, thereby suppressing EMT (Song et al., 2018). USP27X contributes to Snail1 deubiquitination and stabilization, and transforming growth factor-β (TGF-β) is able to promote EMT by upregulating USP27X (Lambies et al., 2019). Meanwhile, TGF-β boosts EMT by upregulating ERK and facilitates phosphorylation of SMAD-specific E3 ubiquitin protein ligase 1 (Smurf1), which, in turn, increases Smurf1-mediated polyubiquitination and degradation of RhoA and disrupts cellular adhesion junctions (Zheng et al., 2022). However, the pro-EMT effect of TGF-β could be inhibited by SCF^β−TrCP1^ through SMAD4-dependent ubiquitination (Wan et al., 2005).

Aside from TGF-β, the Wnt/β-catenin and Notch signaling pathways also contribute to EMT. USP18 stabilizes Notch receptor 1 (Notch1) by removing K48-linked ubiquitins and upregulating MYC to promote EMT (Feng et al., 2020). We have mentioned that E3 and DUBs modulate the Wnt/β-catenin pathway, while their effects in the EMT process remain to be further explored.

In addition, some E3s or DUBs contribute to EMT by mediating some transcription factors. Skp2 promotes EMT by targeting MYC and recruiting E1A-binding protein p300 to the enhancer region of Zeb1, a key EMT activator functioning on cellular plasticity of PDAC (Krebs et al., 2017), to enhance its transcription (Wang et al., 2022). All these studies indicate the significance of E3s and DUBs in regulating the EMT of PDAC.

### 2.6 E3s and DUBs regulate metastasis in PDAC

Metastasis incidence is the reason for shorter OS in patients with PDAC, and numerous mechanisms and pathways are involved in metastasis, in which the roles of E3s and DUBs cannot be ignored. Capping actin protein of muscle z-line subunit alpha 1 (CAPZA1) can be degraded by UBR5-dependent ubiquitination, which then induces the accumulation of F-actin and metastasis (Li et al., 2021b). USP14 targets TAZ for K48-linked deubiquitination and stabilizes TAZ to promote PDAC liver metastasis in murine models (Zhao et al., 2023). USP33 can promote TGFBP2 deubiquitination and TGF-β activation, which, in turn, targets Zeb1 to trigger the transcription of USP33, thus forming a positive feedback loop to stimulate PDAC metastasis (Liu et al., 2023c). USP5 stabilizes WT1 transcription factor (WT1) by removing ubiquitin molecules, thereby leading to an increase of E-cadherin, while the compound WP1130 suppresses the progression of PDAC by blocking USP5 (Li et al., 2020c).

In addition, YOD1 deubiquitinase is highly expressed in PDAC and accelerates tumor metastasis, although the underlying mechanism remains unclear (Zhang et al., 2022). It is noticeable that USP9X is reported inactivated in more than 50% of PDAC, and its level is inversely associated with metastatic burden in advanced disease, which may result in anoikis (Perez-Mancera et al., 2012) and inhibition of programmed cell death (Taddei et al., 2012).

### 2.7 E3s and DUBs regulate metabolism in PDAC

The rapid proliferation of tumor cells is inevitably accompanied by a high level of intracellular metabolism. Unlike normal tissues, tumors tend to metabolize glucose to lactate via aerobic glycolysis even when sufficient oxygen is available to support mitochondrial oxidative phosphorylation, which is known as the Warburg effect (Warburg, 1956). It has been speculated why tumor cells favor utilizing aerobic glycolysis; the reason is that much more ATP would be produced efficiently in this way compared to oxidative phosphorylation, and this also provides more intermediates for other biosynthesis (DeBerardinis et al., 2008).

The aerobic glycolysis process is also regulated by E3s and DUBs. For instance, UBR5 promotes aerobic glycolysis in PDAC by binding to and accelerating the ubiquitin-mediated degradation of CCAAT enhancer-binding protein alpha (C/EBPα), thereby suppressing fructose-1, 6-biphosphatase (FBP1), which is a rate-limiting enzyme, and preventing its inhibition of aerobic glycolysis (Chen et al., 2021b). TRIM47 also enhances aerobic glycolysis by directly binding to FBP1 and leading to its ubiquitination degradation (Li et al., 2021c). USP10 promotes glycolysis by deubiquitinating and stabilizing phosphoglycerate kinase 1 (PGK1) (Quan et al., 2023), the first ATP-producing enzyme in glycolysis (Nie et al., 2020). Meanwhile, USP25 promotes glycolysis through the stabilization of HIF-1α by mediating its deubiquitination (Nelson et al., 2022). Conversely, FBXW7 facilitates the ubiquitination of MYC and suppresses aerobic glycolysis (Qin et al., 2019).

In addition to glucose metabolism, both amino acid and lipid metabolism play crucial roles in the progression of PDAC. It is found that USP21 promotes tumorigenesis through regulating microtubule affinity-regulating kinase 3 (MARK3)-induced macropinocytosis, thereby maintaining intracellular amino acids used for anabolism (Hou et al., 2021). In addition, branched-chain amino acids (BCAAs) directly participate in protein synthesis, and their degradation products provide essential raw materials for other metabolisms. TRIM21 leads to the ubiquitination degradation of BCAA transaminase 2 (BCAT2), which suppresses BCAA uptake and catabolism, thereby inhibiting the progression of PDAC (Li et al., 2020d).

TRIM15 promotes the polyubiquitination and degradation of apolipoprotein A1 (APOA1), which is involved in lipid transport and metabolism, and PDAC metastasis by enhancing lipid droplet accumulation (Sun et al., 2021b). Altogether, we have seen the crucial capabilities of E3s and DUBs in regulating the three main metabolic pathways of PDAC.

### 2.8 E3s and DUBs regulate TME in PDAC

Emerging studies have revealed that the tumor microenvironment (TME) is crucial for tumor progression and metastasis, the homeostasis of which is balanced by a close crosstalk within and across all cellular compartments, including malignant, endothelial, stromal, and immune cells in it. Meanwhile, inflammation in the TME is generally considered a hallmark of cancer progression (Balkwill and Mantovani, 2001). Among the diverse inflammatory cells infiltrating into the TME, tumor-associated macrophages (TAMs) are of significant importance as they can be polarized into the pro-inflammatory subtype M1 or the anti-inflammatory subtype M2, with M1 inhibiting and M2 promoting the tumor progression (Boutilier and Elsawa, 2021; Wang et al., 2021). Emerging evidence demonstrated that the polarization of TAMs is finely regulated by E3s and DUBs. For instance, deletion of the fragile site-associated tumor suppressor (FATS) in PDAC cells blocks the NF-κB/IκBα loop, thus prolonging the activation of NF-κB and promoting the polarization of TAM from M2 toward M1 (Zhang et al., 2021c). USP10 decreases ubiquitin-mediated degradation of YAP1 and further elevates cysteine-rich 61 (Cyr61), which then restores programmed cell death 1 ligand 1 (PD-L1) and galectin-9 expression and stimulates M2 polarization, thereby helping tumor cells evade from immune surveillance (Liu et al., 2022).

T regulatory cells (Treg) are important components that encourage oncogenesis in the TME and exhibit intrinsic immunosuppressive capabilities, which enable them to maintain immune tolerance inside the body and prevent excessive inflammatory reactions that could cause organ damage. It is reported that the differentiation of Tregs is promoted by USP44 through facilitating the deubiquitination of FOXP3 (Yang et al., 2020a). On the contrary, cytotoxic T lymphocytes (CTLs) play tumor-killing roles in the TME, and it is illustrated to be activated by an application of the USP8 inhibitor along with anti-PD-L1 agents because inhibition of USP8 could prevent PD-L1 from proteasome degradation (Yang et al., 2023). These findings indicate that E3s and DUBs are significant in the modification of the immune response in the TME of PDAC.

## 3 E3s and DUBs regulated by mi/circ/lncRNA

In past decades, there has been a growing recognition of the significance of non-coding RNAs (ncRNAs), including microRNAs (miRNAs), circular RNAs (circRNAs), and long non-coding RNAs (lncRNAs). These ncRNAs not only have a direct impact on the expression of RNA, as represented by miRNAs binding to the 3′untranslated region (3′UTR) of target mRNAs to facilitate mRNA degradation or translation suppression, but also play a role in enhancing protein–protein interactions as scaffolds, and the latter kind of action is particularly noticed on circRNAs and lncRNAs. Consequently, these ncRNAs also contribute to the development of PDAC by influencing the regulation process E3s and DUBs act on.

USP9X is able to suppress tumor proliferation and EMT by activating the Hippo pathway by deubiquitinating and increasing the stability of LATS (Toloczko et al., 2017), and miR-212 downregulates USP9X by attaching to its 3′UTR (Chen et al., 2019). Noting that USP9X suppresses the metastasis of PDAC but promotes GEM resistance, it is easy to speculate that USP9X might recognize and mediate diverse substrates which hold the same domain, thereby inducing various functions. Smurf2 inhibits TGF-β-mediated EMT, while miR-15b downregulates Smurf2 by degrading its mRNA (Zhang et al., 2015).

Regarding scaffold function, lncRNAs perform as a double-edged sword in regulating malignancy. LncSOX21-AS1 encourages tumor stemness and EMT by attracting USP10, thereby promoting the deubiquitination of SRY-box transcription factor 21 (SOX21) (Yu et al., 2022). LncCF129 enhances the invasion and proliferation of PDAC by promoting the binding of Makorin ring finger protein 1 (MKRN1) to P53, thus leading to its ubiquitination degradation (Liu et al., 2019). LINC00623 is able to bind to N-acetyltransferase 10 (NAT10) and recruit USP39, which prevents NAT10 from ubiquitin-mediated degradation, thereby promoting tumor progression by stabilizing the downstream oncogenic mRNAs through mediating the N4-acetylcytidine modification (Feng et al., 2022). LINC00857 also facilitates the metastasis of PDAC by promoting OTUB1-mediated deubiquitination of FOXM1 (Zhang et al., 2023b). Conversely, LncMTSS1-AS inhibits the metastasis of PDAC by enhancing the interaction of stress-induced phosphoprotein 1 (STIP1) and myeloid zinc finger 1 (MZF1), thereby promoting ubiquitin-dependent degradation of MZF1 and further suppressing MZF1-mediated transcription activation of MYC (Hu et al., 2020). LINC00673 is also clarified to suppress tumor progression by enhancing the binding of pre-mRNA processing factor 19 (PRPF19) to protein tyrosine phosphatase non-receptor type 11 (PTPN11), subsequently promoting PRPF19-mediated ubiquitination and degradation of PTPN11 (Zheng et al., 2016).

Apart from their scaffolding capabilities, lncRNAs and circRNAs also serve as molecular sponges, effectively capturing miRNAs and restoring the expression of targets. LINC00976 adsorbs miR-137 to rescue OTUD7B mRNA from degradation, which further enhances the deubiquitination of EGFR to promote the malignance of PDAC via the MAPK pathway (Lei et al., 2019). In addition, the interaction between lncRNAs and some specific protein domains forms a spatial site barrier, which restricts the binding of other proteins to the target site on substrates. For instance, LncSLC7A11-AS1 combines with the F-box domain of beta-transducin repeat containing E3 ubiquitin–protein ligase (β-TRCP1), thus blocking SCF binding and inhibiting the formation of the SCF^β−TRCP^ E3 complex, which could mediate the ubiquitination degradation of Nrf2, thus leading to the downregulation of reactive oxygen species (ROS), finally promoting the stemness of PDAC and GEM resistance (Yang et al., 2020b).

CircRNAs, as an important type of non-coding RNAs, have been widely studied in cancer types, and several research studies have illustrated the function of circRNAs in interacting with E3s or DUBs in PDAC. The ubiquitination of caveolin 1 (CAV1) mediated by zinc and ring finger 1 (ZNRF1) is blocked by circFARP1, thus leading to the release of leukemia inhibitory factor (LIF1) in cancer-associated fibroblasts (CAFs) and eventually promoting GEM resistance (Hu et al., 2022). CircSTX6 also plays a role as a spatial site barrier, which promotes proliferation and metastasis in PDAC through competitive binding with cullin 2 (CUL2) and thus suppresses von Hippel–Lindau (VHL)–CUL2 complex-dependent ubiquitination of HIF-1α (Meng et al., 2022). Despite all this, additional important roles of circRNAs in PDAC still need further exploration.

## 4 E3s and DUBs regulate other biological processes involved in PDAC

**FIGURE 2:**
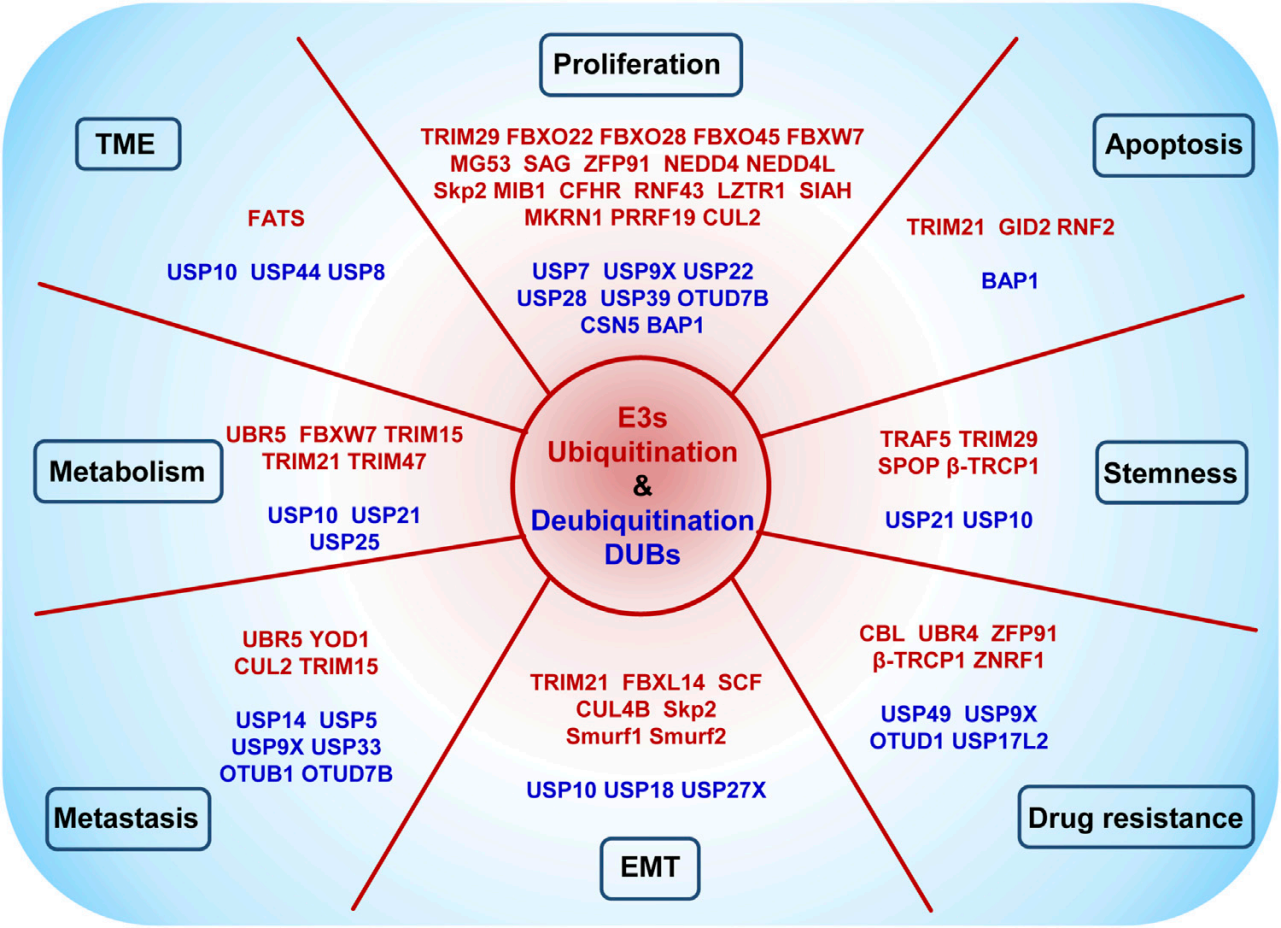
Schematic representation of E3s and DUBs acting on different phenotypes of PDAC.

**FIGURE 3:**
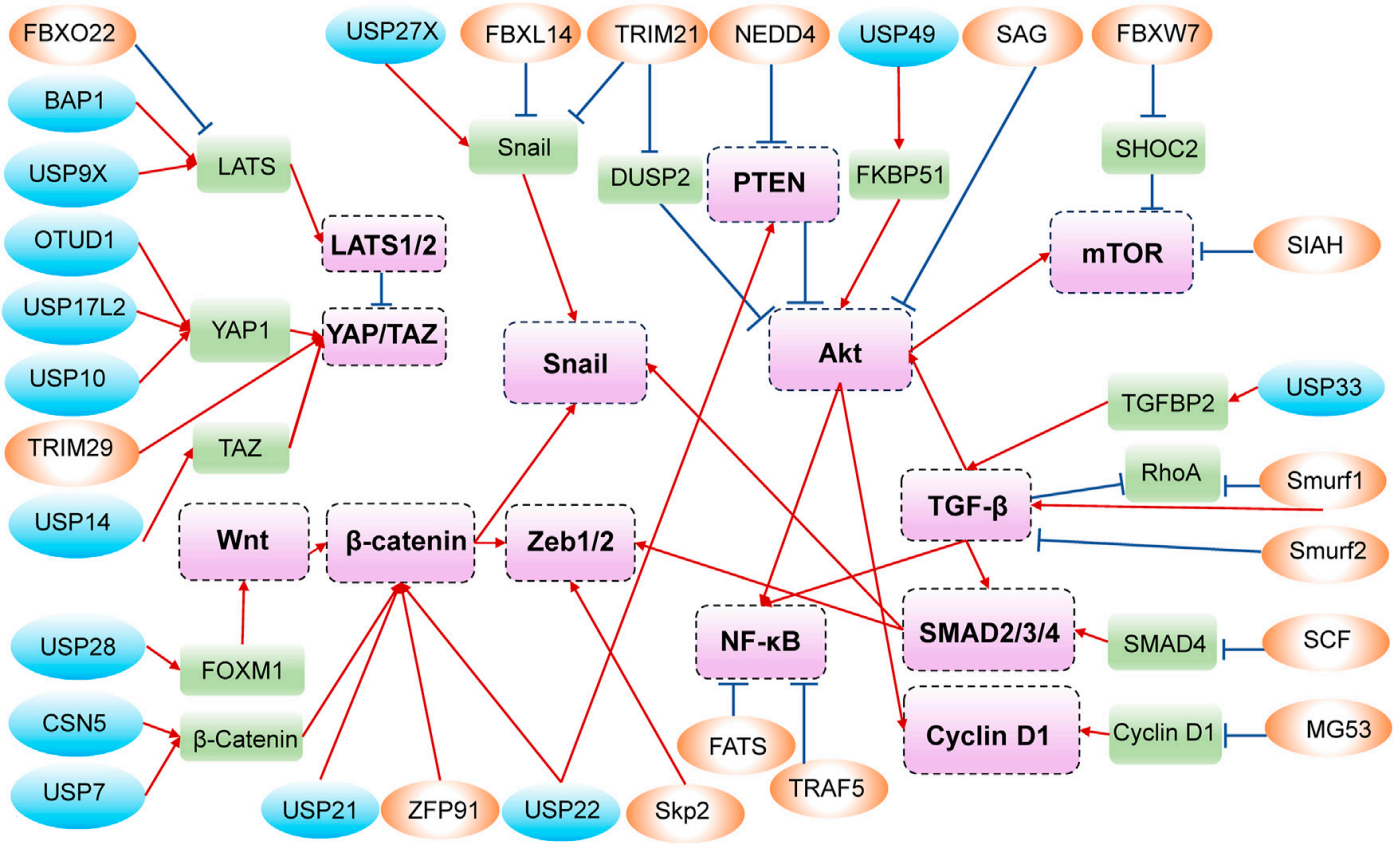
Crosstalk among E3s/DUBs, their dominant substrates, and signaling pathways.

In addition to all the above phenotypes, which are shown in Figure 2, and the crosstalk of related signaling pathways, shown in Figure 3, some unexpected roles of E3s and DUBs have also been discovered in PDAC, including muscle contraction, DNA methylation, and genomic stability. For instance, patients with advanced PDAC would develop cachexia, and UBR2 possibly associates with the development of muscle atrophy in such patients (Wu et al., 2022b). The muscle-specific E3 ubiquitin ligase muscle ring finger 1 (MuRF1) mediates ubiquitination degradation of cytoskeletal and muscle contractile proteins, such as desmin (DES), myosin heavy chain 4 (MYH4), and troponin T3 (TNNT3), and knockdown of MuRF1 in KPC model slows down tumor growth and leads to the accumulation of metabolites, thereby inhibiting PDAC-induced muscle wasting (Neyroud et al., 2023).

Moreover, DNA methyltransferase 1 (DNMT1) can catalyze the methyl group transfer to the CpG island of DNA to mediate DNA methylation and USP7 can induce the deubiquitination of DNMT1, while acetylation of DNMT1 disrupts the function of USP7 and leads to DNMT1 degradation (Cheng et al., 2015). USP7 can also deubiquitinate and block the nuclear localization of FBP1, which is responsible for increasing the sensitivity of PARP inhibitors by interacting with DNMT1 and trapping PARP1 in chromatin (Cheng et al., 2022). In addition, it is reported that DNMT1 promotes tumor angiogenesis (Zhu et al., 2021b) and cell stemness (Zagorac et al., 2016) in PDAC, although whether and how E3s and DUBs regulate these phenotypes still need to be further elucidated.

In addition, apart from suppressing proliferation via the degradation of mTOR, FBXW7 plays a crucial role in maintaining the genomic stability of PDAC through promoting K63-linked polyubiquitination of X-ray repair cross-complementing 4 (XRCC4) to accelerate the formation of the nonhomologous end-joining (NHEJ) complex (Zhang et al., 2016) and the repair of DNA double-stranded breaks (DSBs). Obviously, FBXW7 is an important tumor suppressor E3 ubiquitin ligase.

Annotation: The circles in blue represent DUBs, while those in orange represent E3s. The rounded rectangles in green are substrate proteins, and those in purple are important molecules in signaling pathways. The positive adjustment is represented by red arrows, whereas the negative regulation is shown in blue with a short line at the end.

## 5 Advances in E3- and DUB-dependent PDAC therapy

**FIGURE 4:**
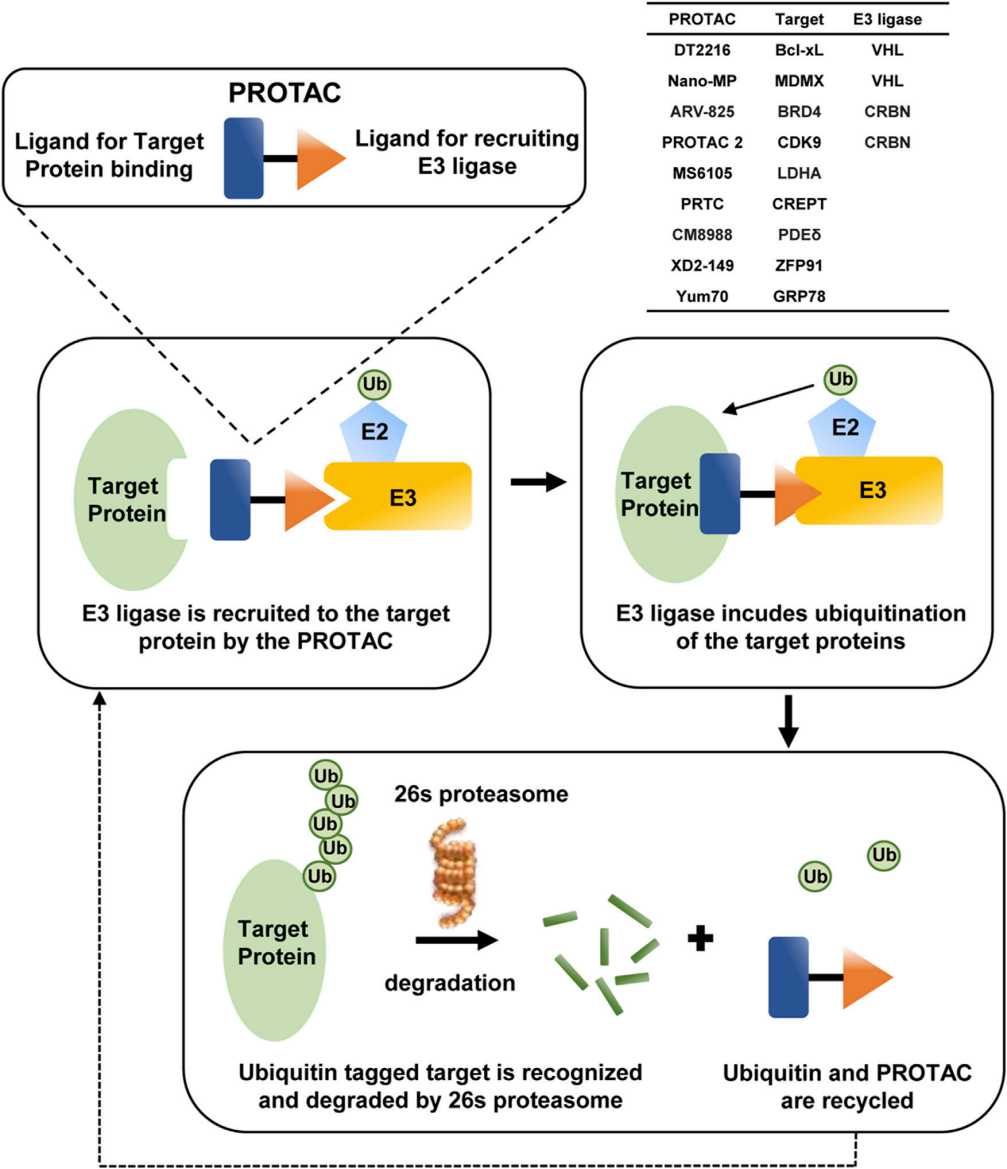
Sketch map of PROTAC-mediated degradation of target proteins in PDAC.

The application of small molecules targeting disease-causing proteins is a traditional technique in cancer therapeutics, and this strategy is improved along with the development of PROTACs from 2001 (Sakamoto et al., 2001), possessing the superiority to kill the distance between the intracellular target proteins and E3 ubiquitin ligases, thereby bringing about the degradation of the targets by ubiquitination more specifically (Bekes et al., 2022). PROTAC is composed of two ligands to the target protein or E3 and a linker connecting the two ligands, and as long as the target-PROTAC-E3 ternary complex is formed, E2 would transfer ubiquitin to target proteins; then the polyubiquitinated substrates would be degraded through proteasome (An and Fu, 2018), the process of which is depicted in Figure 4.

Several PROTACs have been reported to attenuate tumor progression in PDAC. Among them, DT2216 has entered the clinical stage and with the best prospects, which targets B-cell lymphoma/leukemia-xL (Bcl-xL) to the VHL E3 ligase for degradation, thereby inhibiting the anti-apoptotic function of the B-cell lymphoma/leukemia-2 (Bcl-2) family. DT2216 also improves the antitumor efficacy of GEM (Khan et al., 2019; Thummuri et al., 2022) or Sotorasib *in vivo* (Khan et al., 2022), even for PDAC cells with the *Kras* ^*G12D*^ mutation (Khan et al., 2023). In addition, Nano-MP is another PROTAC relying on VHL; it induces the MDM4 regulator of p53 (MDMX) degradation and restores the anti-cancer function of p53 and p73 in PDAC (Yan et al., 2021).

The PROTAC degrader ARV-825, which effectively degrades bromodomain-containing protein 4 (BRD4) via E3 ligase cereblon (CRBN)-mediated ubiquitination, exhibits potent efficacy against several cancer types, including PDAC (Minko, 2020; Saraswat et al., 2020). Another aminopyrazole PROTAC based on CRBN is first proposed to degrade CDK9 and has shown a significant antitumor effect on colorectal carcinoma (Robb et al., 2017). Based on this, PROTAC 2 is developed with improved linker length and composition, which sensitizes PDAC cells to Bcl-2 inhibitor venetoclax more efficaciously through degrading CDK9 and further inactivating Mcl-1 apoptosis regulator (Mcl-1), a Bcl-2 family member (King et al., 2021).

In particular, some PROTACs are only studied in PDAC. MS6105 is the first lactate dehydrogenase (LDH) PROTAC to degrade LDHA (Sun et al., 2023), PRTC specifically targets cell cycle-related and expression-elevated protein in tumor (CREPT) for degradation (Ma et al., 2020), CM8988-PIPD induces phosphodiesterase delta (PDEδ) degradation (Fan et al., 2022), and XD2-149 degrades ZFP91, which promotes tumorigenesis through NF-κB and HIF-1α (Hanafi et al., 2021). YUM70 is known as a small-molecule inhibitor of GRP78, and PROTAC based on it is first synthesized to force the degradation of GRP78 in PDAC (Samanta et al., 2021). All of these PROTACs succeed in *in vitro* experiments, and whether they would achieve ideal therapeutic effects *in vivo* is still under exploration.

PROTACs have shown significant potential for treating cancer in clinical applications. Up to now, more than 10 PROTACs, such as ARV-110, ARV-471, and CFT7455, have been licensed for phase I clinical trials in treating various cancer types. Among them, only DT2216 is reported to inhibit PDAC progression; however, it is only permitted for phase I clinical trials as a treatment for peripheral and cutaneous T-cell lymphoma. This might be explicated that the targeting and degradation activity of PROTACs are distinct in different tumor environments. It makes great sense to explore the more effective tumor targets and more E3s in PDAC, and there is still a long way to go until effective PROTACs are permitted in clinical application.

## 6 Summary and perspectives

The ubiquitin system is complex, multifaceted, and crucial for the modulation of a vast number of cellular processes. This review depicts the landscape of how the ubiquitination and deubiquitination system works on diverse phenotypes of PDAC and visualizes the complex network among E3s, DUBs, substrates, and their regulatory factors reported in PDAC until now, although in-depth interactions of the specific substrates and molecular mechanisms of several enzymes involved in multiple phenotypes still remain unclear. Elucidation of these mechanisms could expand our understanding of the significant roles of E3s and DUBs in PDAC development and would also provide instructive ideas in our subsequent search for PDAC biomarkers and therapeutic approaches.

We notice that several enzymes play contrary roles in PDAC progression in different phenotypes, such as BAP1, TRIM21, USP22, and USP9X, which seems paradoxical. However, this exactly indicates that the inherent regulation in tumors is a complicated process, especially since these core enzymes function through multiple mechanisms, and the delicate balance between them and pro- or anti-tumors is easily disturbed by different contexts and stages of tumors. Undoubtedly, the flexibility of these enzymes provides both opportunities and challenges for achieving therapeutic goals.

We still endeavor to deal with the challenges ahead, such as defining novel E3s, DUBs, and targeted substrates, investigating the crosstalk among distinct E3s or DUBs, and decoding the unknown pathways linking ubiquitination with other forms of PTMs and cellular physiological mechanisms. It is also necessary to screen the key E3s or DUBs which play a crucial role in regulating the malignant phenotypes and exposing which factors continuously activate E3s or DUBs and what restrain their functions on different substrates. The valuable E3s or DUBs would be promising clinical prognostic indexes and drug targets. PROTAC is a potential therapy strategy by exploiting the intracellular Ub-proteasome system to degrade target proteins, and finding out essential E3s during tumor progression to establish an effective PROTAC for treatment of PDAC is something that benefits and deserves further research.

## Figures and Tables

**Table:** 

**PDAC**	Pancreatic ductal adenocarcinoma
**PTMs**	Post-translational modifications
**E3s**	E3 ubiquitin ligases
**DUBs**	Deubiquitylases
**PROTACs**	Proteolysis-targeting chimeras
**Ub**	Ubiquitin
**Met**	Methionine
**Gly**	Glycine
**GTP**	Guanosine triphosphate
**RNF43**	Ring finger protein 43
**KPC**	LSL-*Kras* ^*(+/G12D)*^LSL-*Trp53* ^*(+/R172H)*^ *Pdx1-Cre*
**LZTR1**	Leucine-zipper-like transcription regulator 1
**SINA**	Seven in absentia
**SIAH**	Seven in absentia homolog
**MST**	Mammalian ste20-like kinase
**LATS**	Large tumor suppressor
**YAP**	Yes1-associated protein
**TAZ**	Tafazzin
**TRIM29**	Tripartite motif-containing 29
**FBXO22**	F-box protein 22
**EMT**	Epithelial–mesenchymal transition
**USP7**	Ubiquitin-specific peptidase 7
**CSN5**	COP9 signalosome subunit 5
**FOXM1**	Forkhead box M1
**MG53**	Mitsugumin 53
**SAG**	S-antigen visual arrestin
**Nrf2**	Nuclear factor erythroid 2-related factor 2
**ZFP91**	Zinc finger protein 91
**NEDD4**	Neural precursor cell-expressed developmentally downregulated 4-like protein
**CDKN1B**	Cyclin-dependent kinase inhibitor 1B
**MIB1**	Mindbomb homolog 1
**ST7**	Suppression of tumorigenicity 7
**CFHR**	Complement factor H-related
**PLK1**	Polo-like kinase 1
**BAP1**	BRCA1-associated protein 1
**PDX**	Patient-derived tumor xenograft
**DUSP2**	Dual-specificity phosphatase 2
**GID2**	Degradation-deficient subunit 2
**RNF2**	Ring finger protein 2
**FBXW7**	F-box and WD repeat domain-containing 7
**LTF**	Lactotransferrin
**TRAF5**	TNF receptor-associated factor 5
**SPOP**	BTB/POZ protein
**TCF7**	Transcription factor 7
**ISG15**	Interferon-stimulated gene 15
**CUL4B**	Cullin 4B
**SIRT1**	Sirtuin 1
**GEM**	Gemcitabine
**UBR4**	Recombinant ubiquitin protein ligase E3 component n-recognin 4
**EZH2**	Enhancer of zeste homolog 2
**HAT1**	Histone acetyltransferase 1
**FKBP51**	FK506-binding protein 51
**OTUD1**	OTU domain-containing protein 1
**OS**	Overall survival
**EGFR**	Epidermal growth factor receptor
**CBL**	Casitas B-lineage lymphoma
**FBXL14**	F-box and leucine-rich repeat protein 14
**TGF-β**	Transforming growth factor-β
**Smurf1**	SMAD-specific E3 ubiquitin–protein ligase 1
**Notch1**	Notch receptor 1
**CAPZA1**	Capping actin protein of muscle z-line subunit alpha 1
**WT1**	WT1 transcription factor
**C/EBPα**	CCAAT enhancer-binding protein alpha
**FBP1**	Fructose-1, 6-biphosphatase
**PGK1**	Phosphoglycerate kinase 1
**MARK3**	Microtubule affinity-regulating kinase 3
**BCAA**	Branched-chain amino acid
**BCAT2**	BCAA transaminase 2
**APOA1**	Apolipoprotein A1
**TME**	Tumor microenvironment
**TAMs**	Tumor-associated macrophages
**FATS**	Fragile site-associated tumor suppressor
**Cyr61**	Cysteine-rich 61
**PD-L1**	Programmed cell death 1 ligand 1
**Treg**	T regulatory cells
**CTLs**	Cytotoxic T lymphocytes
**ncRNAs**	Non-coding RNAs
**miRNAs**	MicroRNAs
**circRNAs**	Circular RNAs
**3′UTR**	3′ untranslated region
**SOX21**	SRY-box transcription factor 21
**MKRN1**	Makorin ring finger protein 1
**NAT10**	N-acetyltransferase 10
**STIP1**	Stress-induced phosphoprotein 1
**MZF1**	Myeloid zinc finger 1
**PRPF19**	Pre-mRNA processing factor 19
**PTPN11**	Protein tyrosine phosphatase non-receptor type 11
**β-TRCP1**	Beta-transducin repeat-containing E3 ubiquitin–protein ligase
**ROS**	Reactive oxygen species
**CAV1**	Caveolin 1
**ZNRF1**	Zinc and ring finger 1
**LIF1**	Leukemia inhibitory factor
**CAFs**	Cancer-associated fibroblasts
**CUL2**	Cullin 2
**VHL**	Von Hippel–Lindau
**MuRF1**	Muscle-specific E3 ubiquitin ligase muscle ring finger 1
**DES**	Desmin
**MYH4**	Myosin heavy chain 4
**TNNT3**	Troponin T3
**DNMT1**	DNA methyltransferase 1
**XRCC4**	X-ray repair cross-complementing 4
**NHEJ**	Nonhomologous end-joining
**DSBs**	DNA double-strand breaks
**Bcl-xL**	B-cell lymphoma/leukemia-xL
**Bcl-2**	B-cell lymphoma/leukemia-2
**MDMX**	MDM4 regulator of p53
**BRD4**	Bromodomain-containing protein 4
**CRBN**	Cereblon
**LDH**	Lactate dehydrogenase
**CREPT**	Cell cycle-related and expression-elevated protein in tumor
**PDEδ**	Phosphodiesterase delta
